# Complete genome sequence of multidrug-resistant and NDM-producing *Klebsiella variicola* clinical isolates

**DOI:** 10.1128/mra.00977-24

**Published:** 2024-12-10

**Authors:** Alejandro Alvarado-Delgado, Neli Nava-Dominguez, Nadia Rodriguez-Medina, Alejandro Sánchez-Pérez, Diana Ortiz-Gutierrez, Rayo Morfin-Otero, Eduardo Rodriguez-Noriega, Federico Alonso Zumaya-Estrada, Ulises Garza-Ramos

**Affiliations:** 1 Instituto Nacional de Salud Pública (INSP), Centro de Investigación sobre Enfermedades Infecciosas (CISEI), Laboratorio de Resistencia Bacteriana, Cuernavaca, Morelos, Mexico; 2 Hospital Civil de Guadalajara (HCG) Fray Antonio Alcalde, Instituto de Patología Infecciosa y Experimental, Universidad de Guadalajara, Guadalajara, Jalisco, Mexico; The University of Arizona, Tucson, Arizona, USA

**Keywords:** *Klebsiella pneumoniae* species complex, carbapenem-resistant, classical phenotype

## Abstract

Recently, global dissemination of NDM-producing *Klebsiella variicola* in hospital settings and natural environments has been described. This study described the whole-genome sequencing of multidrug-resistant phenotype and NDM-producing *Klebsiella variicola* clinical isolates.

## ANNOUNCEMENT

*Klebsiella variicola* has been described as a pathogen in humans (1) due to an increase in its antimicrobial resistance (2) and virulence profiles (3). *K. variicola* is an opportunistic pathogen that has been associated with urinary tract infections (4), bacteremia (5), and occasional outbreaks in pediatric patients (6, 7). In 2018, our laboratory received 321 presumptive *K. pneumoniae* isolates from 7 hospitals in the country. All isolates were recovered in Luria Bertoni medium without antibiotic pressure. A total of 95/321 (29.6%) of the isolates were positive to CarbaNP test (8). To these isolates, PCR-multiplex (9) was applied and two (2.1%) *K. variicola* isolates were identified, and the rest corresponded to *K. pneumoniae* (97.9%).

The two single colonies of *K. variicola* 14876 and 15549 isolates were grown on LB agar and then inoculated in LB broth at 37°C with shaking at 200 rpm overnight. Total genomic DNA was extracted using a DNeasy kit (Qiagen, Germany). Both isolates were sequenced on standard flow cells (2 × 150) paired-end sequencing on Illumina MiSeq libraries (Illumina, Inc. San Diego, CA, USA) with the Nextera XT Sample Prep Kit (Illumina, Inc.). FastQC software v0.11.8 (https://www.bioinformatics.babraham.ac.uk/projects/fastqc/) was used for Illumina reads quality verification and trimmed with Trim_Galore v0.6.4 (https://www.bioinformatics.babraham.ac.uk/projects/trim_galore/). SPAdes Genome Assembler (v3.6.0) software (10) was used for *de novo* assembly of *K. variicola* genomes. The *K. variicola* 14876 and 15549 genomes were annotated using the National Center for Biotechnology Information (NCBI) Prokaryotic Genome Annotation Pipeline (PGAP) (v4.2.0) (https://www.ncbi.nlm.nih.gov/genome/annotation_prok/) (11). Reads longer than 500 bp with 99.33% (14876) and 99.00% (15549) above Q40 were used for *de novo* assembly. In the *K. variicola* 14876 and 15549 genomes, respectively, the total sequence data were 25,960,356 and 23,729,713 reads and a total of 100 and 123 contigs, with an estimated genome size of 5,975, 975bp and 6,076, 063bp with 651× and 585× coverage.

According to the disk-diffusion method (8), the isolates showed resistance to ampicillin, piperacillin, cefotaxime, imipenem, aztreonam, and tetracycline and intermediate susceptibility to ceftazidime and ciprofloxacin, which characterize the isolates as having a multidrug-resistant phenotype. The string test evaluating the hypermucoviscous phenotype in MacConkey agar plates revealed a negative result (12). Table 1 shows the clinical, epidemiological, and genome characteristics of the *K. variicola* isolates. Sequence type (ST) was assigned using the MLST typing tool for *K. variicola* (https://mlstkv.insp.mx) (13). The *K. variicola* 14876 and 15549 isolates were determined as ST114 and ST284, respectively. Kleborate v3.0.8 program was used for capsular serotype determination (14) (https://github.com/klebgenomics/Kleborate?tab=readme-ov-file). Capsular serotypes KL43 and KL39 were identified for *K. variicola* 14876 and 15549 genomes, respectively. Carriage of antimicrobial resistance was determined by the Center for Genomic Epidemiology (15) and virulence genes by BLASTx. The LEN-17 and LEN-19 chromosomal β-lactam resistance gene was identified in *K. variicola* 14876 and 15549 genomes, respectively. In addition, the resistome of the 14876 genome showed *aadA16, aadA2, aph(3')-Via*, *qnrA1*, *arr-3, sul1, dfrA27,* CARB-2, and NDM-1; the 15549 genome showed *rmtC*, *sul1,* and NDM-1. Virulence genes found in 14876 and 15549 genomes were the siderophore and ferric-acquisition ABC (ATP-Binding-Cassette) transporter *kfuABC* that is utilized for iron uptake (16) as well as the siderophore enterobactin *ent* operon (17). The type 3 fimbriae *mrkABCDFHIJ* operon and *ureABDFG* operon were also identified. Plasmid incompatibility groups (Inc) were detected by PlasmidFinder-V2.1 (18) for the *K. variicola* 14876 and 15549 genomes. The Inc identified are described in Table 1.

**TABLE 1:** Clinical, epidemiological, and genome data of NDM-producing *K. variicola* clinical isolates

Characteristic	*K. variicola* isolates	
Strains	14876	15549
Origin	Bloodstream	Intra-abdominal secretion
Year of isolation	2018	2017
Sex	Male	Male
Symptomatic or asymptomatic	Symptomatic	Symptomatic
Susceptibility profile	Multidrug-resistant phenotype	Multidrug-resistant phenotype
Genomic		
Protein coding	5,653	5,798
tRNA	76	77
rRNA	16	16
C + G content (%)	57.25	57.28
Total size (bp)	5,975,975	6,076,063
N50	385054	328675
Number of contigs	100	123
GenBank accession no.	JBGLNC010000000	JBGLNB010000000
SRA database	SRR30842875	SRR30889203
Genetic characteristics		
Sequence type^*a*^	114	284
Capsular serotype^*b*^	KL43	KL39
Resistome^*c*^	LEN-17*, aadA16, aadA2, aph(3')-Via, qnrA1, arr-3, sul1, dfrA27,* CARB-2, NDM-1	LEN-19*, rmtC*, *sul1,* NDM-1
Virulome^*d*^	*kfuABC, entABC, mrKABCDFHIJ, ureABDFG,*	*kfuABC, entABC, mrKABCDFHIJ, ureABDFG*
Incompatibility group^*e*^	FIA(pBK30683), IncFII(K), IncFIB(K), IncA/C2	IncFIA(pBK30683), IncFIB(K), IncFIB(pKPHS1), IncFII(K), IncFII(Yp)

^*a*^ Sequence type determined by MLST of K. variicola.

^*b*^ Capsular serotype determined by Kleborate.

^*c*^ Resistome determined by Center for Genomic Epidemiology.

^*d*^ Data base of virulence factor of K. pneumoniae species complex used by identification by BLASTx.

^*e*^ Incompatibility group determined by Center for Genomic Epidemiology.

## Data Availability

This whole-genome shotgun project has been deposited in the BioProject as PRJNA1148126, and GenBank and SRA with accession numbers JBGLNB000000000 and SRR30889203 for *Klebsiella variicola* 15549 and JBGLNC000000000 and SRR30842875 for *Klebsiella variicola* 14876, respectively.
